# Resting-state fMRI study on drug-naive patients of essential tremor with and without head tremor

**DOI:** 10.1038/s41598-018-28778-z

**Published:** 2018-07-12

**Authors:** Ling Wang, Du Lei, Xueling Suo, Nannan Li, Zhongjiao Lu, Junying Li, Jiaxin Peng, Qiyong Gong, Rong Peng

**Affiliations:** 1 Department of Neurology, West China Hospital, Sichuan University, Sichuan, 610041 China; 2 Huaxi MR Research Center (HMRRC), Department of Radiology, West China Hospital, Sichuan University, Sichuan, 610041 China

## Abstract

This study used resting-state functional MRI (r-fMRI) to evaluate intrinsic brain activity in drug-naive patients with essential tremor (ET) with and without head tremor. We enrolled 20 patients with ET with hand and head tremor (h-ET), 27 patients with ET without head tremor (a-ET), and 27 healthy controls (HCs). All participants underwent r-fMRI scans on a 3-T MR system. The amplitude of low-frequency fluctuation (ALFF) of blood oxygen level-dependent signals was used to characterize regional cerebral function. We identified increased ALFF value in the bilateral posterior lobe of cerebellum in the h-ET patients relative to a-ET and HCs and demonstrated that h-ET is related to abnormalities in the cerebello-cortical areas, while the a-ET is related to abnormalities in the thalamo-cortical areas. In addition, we observed the ALFF abnormality in the cerebellum (left cerebellum VIII and right cerebellum VI) correlated with the tremor score in h-ET patients and abnormal ALFF in the left precentral gyrus correlated with the age at onset and disease duration in h-ET patients. These findings may be helpful for facilitating further understanding of the potential mechanisms underlying different subtypes of ET.

## Introduction

Essential tremor (ET) is one of the most common neurodegenerative disease, and is characterized by progressive postural and kinetic tremor affecting the hands, head, or other parts of the body^1^. Currently, ET is also known to be a clinically heterogeneous disorder involving many other non-motor symptoms, such as cognitive, psychiatric, dementia and sensory impairment^2^. The worldwide prevalence is about 1%, increasing to approximately 5% of the population over 65 years of age^3^. Despite its high prevalence, the pathophysiology of ET is still poorly understood.

Head tremor (HT) is present in nearly 35% to 48% of the ET patients^4–7^. Several demographic and clinical studies find ET patients with HT may be older, more likely to be female, had an older age of onset than the patients without HT, suggesting that patients with HT have a distinct clinical phenotype and may be a subtype of ET^7,8^. In addition, previous imaging studies have shown ET patients with and without HT differ. With the aid of structural neuroimaging techniques, grey matter loss in the cerebellar vermis and entire cerebellum, were detected in ET patients with HT^9,10^. These findings provide a structural basis for ET patients with HT.

Resting-state functional MRI (r-fMRI), which utilizes blood oxygen level-dependent (BOLD) signals to determine regions of activity, provides an important non-invasive method to assess brain regional and neural circuitry function. Besides, this method requires minimal patient compliance, avoids potential confounds associated with variable tasks, and is relatively easy to implement in clinical studies^11,12^. Thus, the r-fMRI enables the examination of synchronous regional cerebral activity alterations by using the amplitude of low-frequency fluctuation (ALFF) analysis. To our knowledge, to date, there are 2 studies using ALFF to detect the intrinsic, spontaneous brain activity in ET patients, and proposing disturbances in the low frequency oscillation in the cerebellar–motor cortical circuit in patients with ET^13,14^. However, few report has applied ALFF in HT patients study.

This investigation aims to use r-fMRI to characterize changes in regional ALFF intensity of the brain network in drug-naive patients with ET with and without head tremor, compared with a cohort of healthy controls. Then, several regions were defined based on the group differences, followed by correlation analyses between the ALFF value in those regions and ET clinical assessments. This investigation may provide an explanation for clinical differences between ET patients with and without HT.

## Results

### Demographic and clinical characteristics

Finally, 47 patients with ET were grouped according to the presence or absence of head tremor. 27 patients with ET had arm ET (a-ET; 16 males, 11 females, mean age 45.00 ± 14.43), whereas the remaining 20 patients had both a-ET and head ET (h-ET; 7 males, 13 females, mean age 51.00 ± 12.10). Demographic and clinical features of the sample were listed in Table 1. Age, gender, handedness and MMSE score were not significantly different among the three groups. No significant difference in age of onset, disease duration, family history, MMSE and TRS score (TRS- A&B, TRS-C) was found between h-ET and a-ET.

**Table 1:** Demographic and clinical characteristics of the total sample.

Parameter	h-ET	a-ET	control	p value^a^	p value^b^
n	20	27	27	—	
Handedness for writing (R:L)	20:0	27:0	27:0	—	
Gender (female:male)	13:7	11:16	12:15	0.222	0.100
Age (years)	51.00 ± 12.10	45.00 ± 14.43	45.78 ± 14.13	0.295	0.139
Age of onset (years)	36.30 ± 12.87	32.81 ± 14.42		—	0.396
Disease duration (years)	14.70 ± 11.58	12.22 ± 8.65		—	0.405
Family history	12	13		—	0.421
Fahn-Tolosa-Marin Tremor Rating Scale (TRS)	18.05 ± 14.54	17.59 ± 13.37		—	0.912
TRS-A&B	15.10 ± 10.83	14.67 ± 10.08		—	0.888
TRS-C	2.95 ± 4.14	3.26 ± 4.11		—	0.800
Medication	0	0		—	—
MMSE score	28.45 ± 1.88	28.63 ± 1.71	28.04 ± 1.68	0.448	—

ET, essential tremor; h-ET, ET patients with both hand and head tremor; a-ET, ET patients with hand tremor but without head tremor; MMSE, Mini-Mental State Exam.

^a^Comparison among h-ET, a-ET patients, and control subjects.

^b^Comparison between h-ET and a-ET patients.

### Comparison of the h-ET and control groups

**Figure 1:**
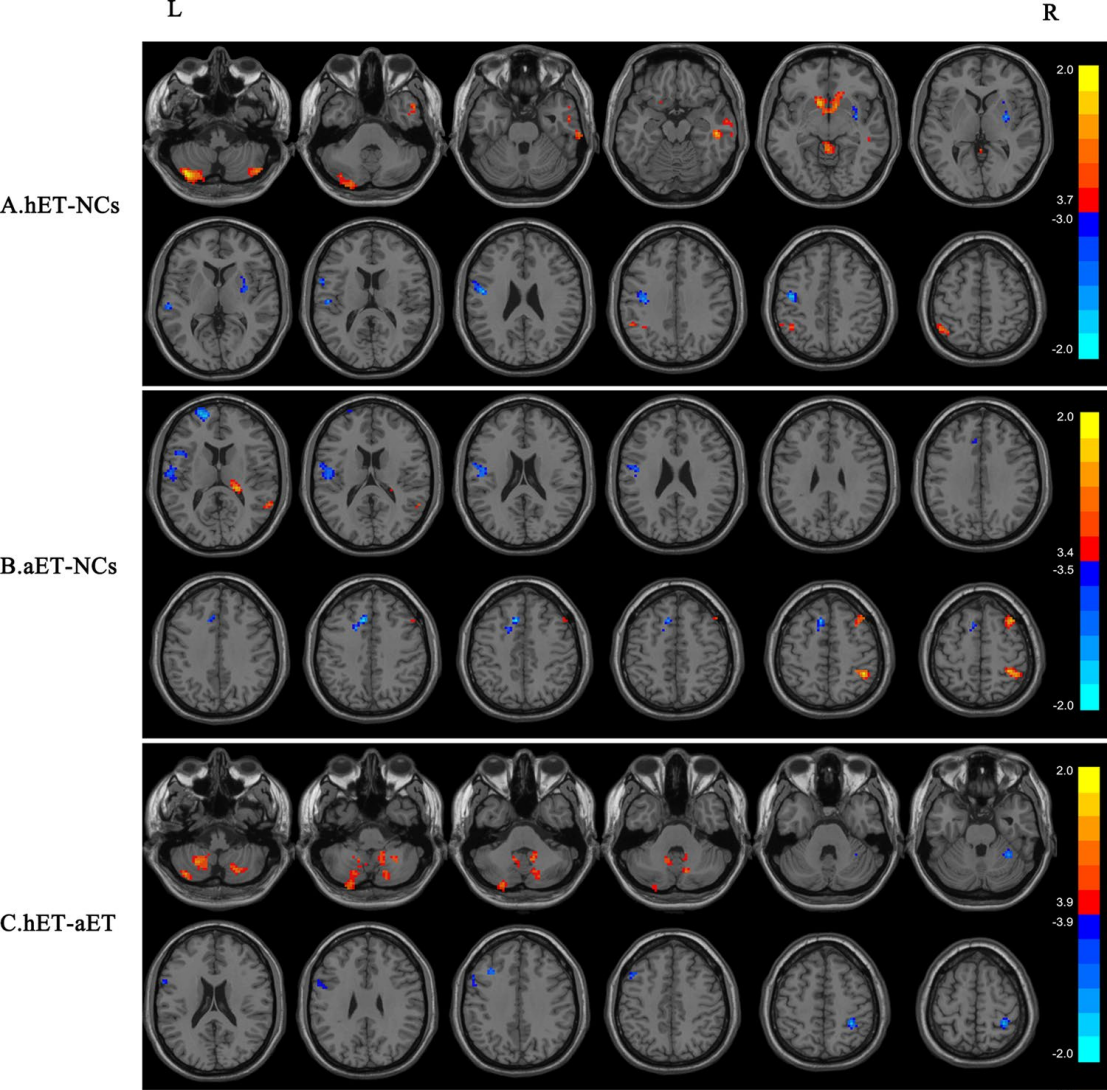
Statistical parametric map showing the significant differences in the ALFF between the h-ET patients, a-ET patients, and HCs. (**A**) The differences between h-ET patients and HCs (the ALFF h-ET < HCs results are in blue, the ALFF h-ET > HCs results are in red). The cluster size >45 voxels. (**B**) The differences between a-ET patients and HCs (the ALFF a-ET < HCs results are in blue, the ALFF a-ET > HCs results are in red). The cluster size >43 voxels. (**C**) The differences between h-ET patients and a-ET patients (the ALFF h-ET < a-ET results are in blue, the ALFF h-ET > a-ET results are in red). The cluster size >42 voxels. The threshold for display was set to P < 0.001.

Compared with normal controls, h-ET patients showed increased ALFF in bilateral posterior lobe of cerebellum (cerebellum VII/Crus II), the upper portion of the cerebellar vermis, bilateral caudate, right middle temporal gyrus and the left inferior parietal lobule. Additionally, decreased ALFF was identified in the right putamen, left precentral gyrus and left postcentral gyrus. The detailed results are shown in Fig. 1 and Table 2.

**Table 2:** Brain regions exhibiting an altered ALFF of the h-ET and a-ET groups compared with the control group.

Brain regions	Cluster size	BA	MNI coordinates			Peak T value
			X	Y	Z	
**h-ET > NCs**						
L cerebellum posterior lobe	309	—	−39	−72	−51	3.738
R cerebellum posterior lobe	59	—	45	−72	−48	3.0478
cerebellum vermis	73	—	0	−45	−6	2.8154
bilateral caudate	167	25	−12	15	−9	3.428
R middle temporal gyrus	160	20	48	−27	−15	3.7251
L inferior parietal lobule	75	40	−48	−54	54	2.6192
**h-ET < NCs**						
R putamen	51	48	30	−3	3	−2.6118
L precentral gyrus	178	6	−42	−15	42	−2.9757
L postcentral gyrus		4				
**a-ET > NCs**						
R thalamus	45	—	21	−30	12	2.938
R middle temporal gyrus	74	22	51	−36	3	3.1893
R middle frontal gyrus	53	9	36	21	60	3.404
R inferior parietal lobule	58	40	42	−45	54	3.2965
**a-ET < NCs**						
L precentral gyrus	141	6	−51	−9	18	−2.6967
L postcentral gyrus		4				
L SMA	78	6	−9	21	42	−3.5012
L insula	56	—	−45	12	0	−2.8374

### Comparison of the a-ET and control groups

The a-ET group demonstrated increased ALFF in the right thalamus, right middle temporal gyrus, right middle frontal gyrus and right inferior parietal lobule. Conversely, the left precentral gyrus, left postcentral gyrus, left supplementary motor area (SMA), and left insula displayed decreased ALFF. The detailed results are shown in Fig. 1 and Table 2.

### Comparison of the h-ET and a-ET groups

Several clusters exhibited significant differences between the h-ET and a-ET groups. Bilateral cerebellum VIII and left CrusII displayed increased ALFF. However, right anterior lobe of cerebellum, left middle frontal gyrus, right postcentral gyrus and right superior parietal lobule displayed decreased ALFF. The detailed results are shown in Fig. 1 and Table 3.

**Table 3:** Brain regions exhibiting an altered ALFF of the h-ET group compared with the a-ET group.

Brain regions	Cluster size	BA	MNI coordinates			Peak T value
			X	Y	Z	
**h-ET > a-ET**						
L cerebellum VIII	159	—	−27	−57	−48	2.9646
R cerebellum VIII	113	—	12	−54	−39	2.7097
L cerebellum Crus II	74	—	−39	−69	−51	3.8422
**h-ET < a-ET**						
R cerebellum VI	80	—	33	−48	−24	−3.4768
L middle frontal gyrus	88	46	−33	24	39	−3.213
R postcentral gyrus	54	3/4	33	−42	57	−3.8964
R superior parietal lobule		40				

### Correlation analyses

**Figure 2:**
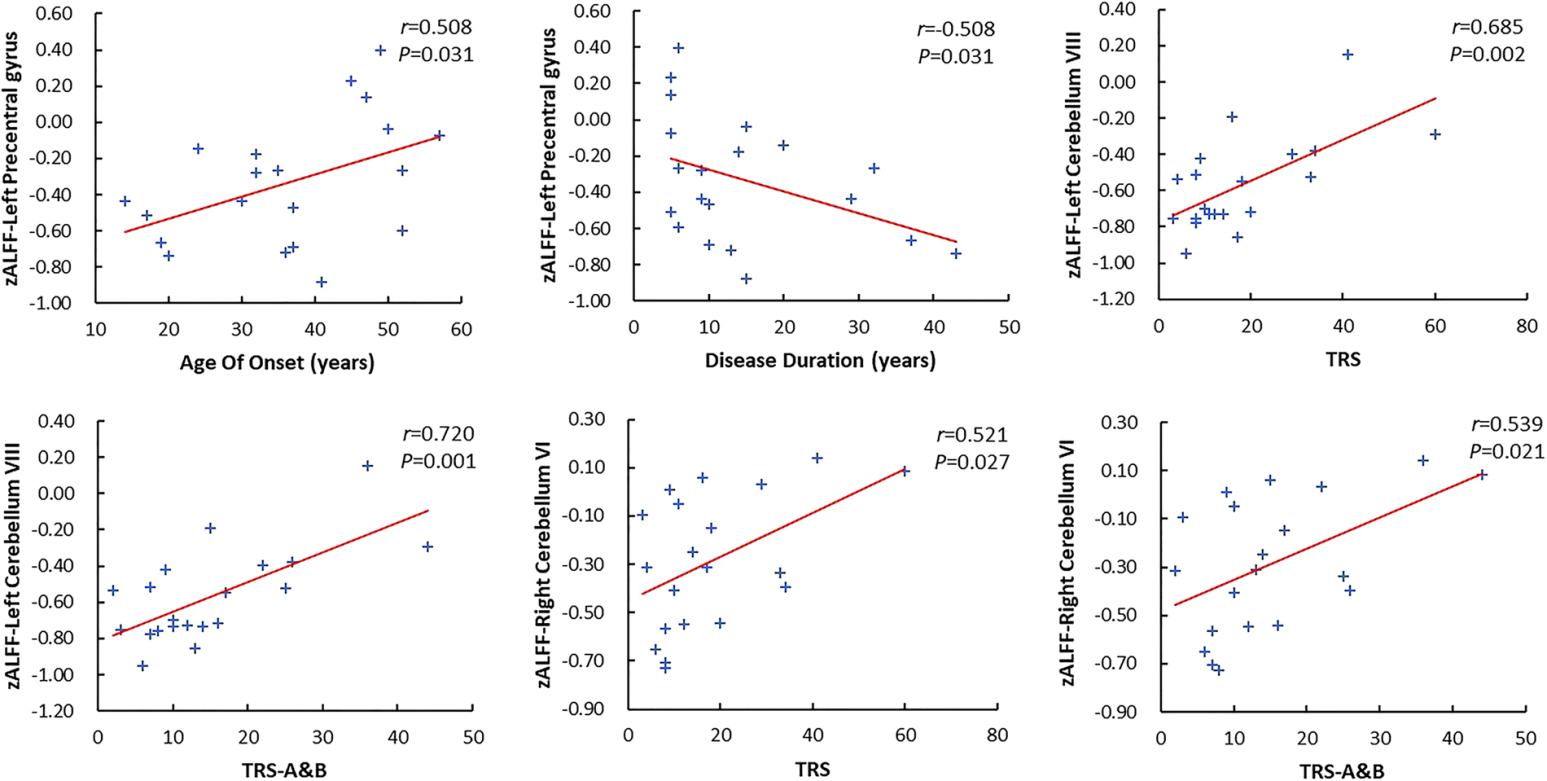
Significantly correlation between ALFF values of regions where the three groups showed significant differences and the clinical indices in patients with h-ET. zALFF: Fisher’s z-transformation of ALFF.

After controlling for the effects of gender and age, a positive correlation with age of onset (r = 0.508, P = 0.031) and a negative correlation with disease duration (r = −0.508, P = 0.031) were observed in the left precentral gyrus in the h-ET patients. Additionally, the ALFF values in the left cerebellum VIII were significantly positively correlated with the TRS (r = 0.685, P = 0.002) and TRS-A&B (r = 0.720, P = 0.001) in the h-ET patients. Besides, a positive correlation with the TRS (r = 0.521, P = 0.027) and TRS- A&B (r = 0.539, P = 0.021) was observed in the right cerebellum VI in the h-ET patients (Fig. 2).

## Discussion

To provide *in vivo* evidence of intrinsic brain abnormalities associated with different characteristics of ET patients with and without head tremor, we calculated the ALFF value of the r-fMRI, a method that has proven to be effective and reliable in reflecting neural activity^15^. The main findings are that h-ET showed increased ALFF value in the bilateral posterior lobe of cerebellum relative to a-ET and HCs, and h-ET and a-ET patients displayed different motor circuit compared to HCs.

### Functional changes in h-ET

The h-ET patients exhibited significantly increased ALFF values in the bilateral cerebellar posterior lobe, cerebellar vermis, bilateral caudate, right middle temporal gyrus and the left inferior parietal lobule. However, decreased ALFF was identified in left primary motor cortex including precentral gyrus and postcentral gyrus.

Previous clinical, electrophysiological, pathological and neuroimaging studies have emphasized the important role of the cerebellum in ET^16–19^. In 2009, using automated volumetric method (FreeSurfer), Cerasa *et al*. found the cerebellum atrophy occurring only in the h-ET patients^9^. Subsequently, in a voxel-based morphometry (VBM) study, Quattrone *et al*. showed the h-ET patients were associated with anterior cerebellar lobe atrophy mainly at the level of the vermis atrophy with respect to healthy controls^10^. Consistent with aforementioned studies, only our h-ET patients showed abnormal brain activity in the bilateral cerebellar posterior lobe and cerebellar vermis. Another study reported that ET patients have altered ALFF during the resting state in the bilateral cerebellum, as well as a significant negative correlation with the duration of tremor^13^. Besides, Passamonti *et al*. observed the increased activation in the cerebellum (crus I/lobule VI) during the execution of high-load working memory trials^20^. By using independent component analysis, in combination with a “dual regression” technique, Benito-Leon *et al*. showed decreased connectivity in the cerebellum and visual networks^21^. In recently studies, Mueller *et al*. found the ET patients had lower general connectivity in the cerebellum and Lenka *et al*. revealed significant changes in the cerebello-thalamo-cortical network in patients with ET, supporting the previous evidence of cerebellar pathology in ET^22,23^. So far, the diffuse cerebellar dysfunction is associated with the heterogeneous symptoms in ET, including motor (from lobule I–V) and cognitive lobules (from lobule VI–IX, including crus I–II). However, the specific relationship between areas of the cerebellum and the clinical phenotypes is still lacking^17^. Our study confirmed previous findings and reinforces the point of the major role of the cerebellum in the pathophysiology of ET.

Structural abnormalities in the prefrontal, parietal and temporal lobes (fronto-temporal cortex) have been shown to be implicated in ET^17^. Our study found decreased ALFF in the right middle temporal gyrus and the left inferior parietal lobule. An fMRI study demonstrated that ET patients displayed overactivation of the dorsolateral prefrontal cortex and the inferior parietal cortex with respect to controls during a Stroop task, suggesting that dysfunctions in the parieto-prefrontal cortices may play a key role in cognitive deficits^24^. Besides, Benito-Leon *et al*. showed increased connectivity in default mode network and frontoparietal networks was associated with cognitive impairment and depressive symptoms^21^. Our findings are consistent with the results of the fMRI studies by Fang *et al*., which also reported that the disruption of local brain activity in the fronto-temporal cortex may be related to the non-motor symptoms, such as cognitive, psychiatric and sensory impairment^25–27^. However, our ET patients have no dementia, and we didn’t evaluate whether our patients have depression and anxiety. Therefore, we proposed that increased ALFF in these areas may act as a compensatory mechanism which prevents ET patients from cognitive symptoms and other non-motor symptoms. We also observed increased ALFF value in the bilateral head of caudate and decreased ALFF value in the right putamen. A previous VBM study demonstrated that brain volumes of the ET group were significantly smaller in many brain regions than that of control group, including the caudate body. They suggested that the resting tremor of ET patients might be due to gradual damage to the BTC (the basal ganglia-thalamocortical) loop^28^. Besides, Brooks *et al*. found that ET patients with resting tremor showed reduced putamen 18F-dopa uptake^29^. This may imply that the ET and Parkinson’s disease (PD) partially share the damage mechanism in BTC loops, which might explain their similar symptoms, such as resting tremor.

Besides, h-ET patients demonstrated decreased ALFF values in left primary motor cortex including precentral gyrus and postcentral gyrus. We observed increased ALFF values in the cerebellum and decreased ALFF values in the cerebral motor cortex seem contradictory. Our findings are consistent with a previous fMRI study which exhibited decreased functional connectivity in the cerebellum network and increased functional connectivity in the sensorimotor and salience networks^25^. We presumed that opposing alterations between motor-cortical and cerebellar ALFF may be under a compensatory hypothesis.

### Functional changes in a-ET

Compared with the healthy controls, the a-ET patients exhibited increased ALFF in the right thalamus and right fronto-temporal areas (middle frontal gyrus, inferior parietal lobule and middle temporal gyrus). Similar to the h-ET patients, the left cerebral cortex that were related to brain regions that are involved in motor function, including the left precentral and postcentral gyrus, left supplementary motor area (SMA), and left insula displayed decreased ALFF. Previous diffusion tensor imaging (DTI) study has revealed significant increase of axial diffusivity in bilateral cerebral hemispheres, thalamus, brainstem and cerebellar hemisphere white matter^30^. Fang *et al*. found two thalamus regions, ventral intermediate (VIM) and mediodorsal (MD) showed decreased regional homogeneity (ReHo), suggesting that thalamus not only involved in the motor dysfunction but also in the non-motor dysfunctions^27^. Consistent with some of previous structural and functional studies, we didn’t find abnormal changes in the cerebellum, however, the reason is unclear now^24,31–34^. Our results may also suggest abnormalities in the thalamo-cortical pathway in a-ET patients.

### Significant difference in the functional changes in h-ET and a-ET

When we directly compared the h-ET and a-ET groups, we found that the h-ET patients showed increased ALFF in the posterior lobe of cerebellum including bilateral cerebellum VIII and left cerebellum crus II and decreased ALFF in the cerebellum VI, left middle frontal gyrus, right postcentral gyrus and right superior parietal lobule in comparison to a-ET patients. Consistent with aforementioned studies^9,10^, only our h-ET patients showed abnormal brain activity in the bilateral cerebellar posterior lobe and cerebellar vermis, whereas these cerebellar regions in a-ET patients did not significantly differ from the controls. According to the hypothesis of cerebellar somatotopic organization, head and neck regions are probably located in the midline portion of the anterior lobe (mainly vermis lobule IV–V), however the hands and legs are located in the paravermis and the cerebellar hemispheres^35^. Then a post-mortem study which found the existence of axonal torpedoes in the cerebellar vermis in ET patients who presented tremors of the neck, voice and jaw confirmed the hypothesis^36^. Furthermore, consistent with our hypothesis, the h-ET and a-ET groups have differences in the motor circuit. Our results indicate that abnormalities in motor circuits may represent the main difference between h-ET and a-ET.

### Correlation Analyses

A previous study of Fang *et al*. found that ReHo abnormalities in the cerebellum and left primary motor cortex correlated with the tremor severity score in ET patients^27^. Subsequently, another study found that aberrant ALFF values in the right precentral gyrus and right cerebellum were associated with disease duration^13^. However, we observed the ALFF abnormality in the cerebellum (left cerebellum VIII and right cerebellum VI) correlated with the tremor score in h-ET patients and abnormal ALFF in the left precentral gyrus correlated with the age at onset and disease duration in h-ET patients, supporting the hypothesis that the abnormality of intrinsic activity in the cerebello-cortical cortex pathway could be associated with the motor-related symptoms of ET^37^.

### Limitations

Our study still has several limitations. First, the relatively small size of the study cohort may have limited statistical power to identify less robust effects. Second, the lack of longitudinal study and non-motor data (anxiety, depression and sensory symptoms) in our ET subjects is also a limitation. Third, BOLD signal in fMRI reflects both neuronal activations and global physiological fluctuations, and the physiological low frequency oscillations, respiration, and cardiac pulsation might affect the estimation of ALFF. Multimodal neuroimaging approaches and multicenter cooperation could be able to make the research more representative.

## Conclusion

Using the ALFF of r-fMRI, our study, the first demonstrated that drug-naive h-ET and a-ET patients display different intrinsic brain activities in their brains, which suggests that the h-ET is related to abnormalities in the cerebello-cortical areas and the a-ET is related to abnormalities in the thalamo-cortical pathways. Additionally, our correlation analyses indicated that abnormal ALFF in different regions were associated with motor-related symptoms in ET. These findings suggest that a-ET and h-ET may be distinct clinical subtypes of the same disease. More studies are required to confirm the findings.

## Materials and Methods

### Participants

The subjects were recruited consecutively from the Department of Neurology of the West China Hospital, Sichuan University from February 2015 to March 2016. A total of 53 patients fulfilled the diagnosis of definite ET according to the Movement Disorders Consensus Criteria^11^. From this cohort, patients with history of thyroid dysfunction, cerebrovascular disorders, dementia, stroke, epilepsy, or head injury were excluded. The patients were drug naive at the initial visit. Functional images were acquired at the same day of clinical assessment. Clinical assessment and image acquisition were conducted prior to the initiation of any treatment. After enrolment, the patients were followed-up for at least 1 year to confirm the diagnosis. The severity of the tremor was assessed using the Fahn-Tolosa-Marin Tremor Rating Scale(TRS). Head tremor was coded as present or absent on examination. The hand tremor was observed in all patients. Additionally, 31 controls were healthy volunteers and were generally matched with patients for age, gender, and area of residence. The controls had no history of neurological or psychiatric conditions. None reported having a first-degree or second-degree relative with ET. All subjects were right-handed. Mini-Mental State Examination (MMSE) was used to evaluate cognition.

The Ethics Committee of Sichuan University approved our study protocol, and written informed consent was obtained from all participants. The methods were carried out in accordance with the approved guidelines.

### Data Acquisition and Image Preprocessing

All participants were scanned using a 3T magnetic resonance system (Tim Trio; Siemens Healthineers, Erlangen, Germany) equipped with a 12-channel phased-array head coil. For each participant, we acquired high-resolution T1- and T2-weighted anatomical images, and a radiologist viewed the images to exclude subjects with space-occupying lesions and cerebrovascular diseases. Functional images were obtained using a standard Echo Planar Imaging pulse sequence with the following parameters: repetition time (TR) = 2000 ms; echo time (TE) = 30 ms; flip angle = 90°; slice thickness = 5 mm (no gap); matrix size = 64 × 64; field of view (FOV) = 240 × 240 mm^2^; voxel size = 3.75 × 3.75 × 5 mm^3^; scanning time = 8 minutes. A total of 240 volumes (30 slices per volume) were collected for each subject. The scanning was performed in darkness, and the participants were instructed to keep their head still, relax, close their eyes and think of nothing during the acquisition. Earplugs were used to reduce the scanner noise, and foam pads were used to minimize the head motion.

Image preprocessing was carried out using the Data Processing & Analysis for Brain Imaging (DPABI, http://rfmri.org/dpabi) running on MATLAB R2014a (The Math-Works Inc., Natick, MA, USA)^38^. The first 10-time points were discarded for signal equilibrium and participants’ adaptation to scanning noise. Then, the remaining images were corrected for intravolume acquisition time delay, head motion and spatial normalization (3 × 3 × 3 mm^3^) to the standard Montreal Neurological Institute (MNI) template. The following step was spatial smoothing with 8 mm full-width at half-maximum (FWHM) Gaussian kernel. According to records of head motions with Friston-24 correction using Friston24-parameter model within each fMRI run, we calculated mean and maximum head displacements in each subject and compared it between three groups. The mean and maximum head displacements were not significantly different among the three groups, and 6 patients (4 h-ET patients and 2 a-ET patients) and 4 controls with a maximum displacement during imaging that exceeded 1 mm in any cardinal direction or with a maximum rotation larger than 1° were excluded.

### ALFF Calculation

ALFF map for each subject were calculated by using the DPABI toolbox^38^. Linearly detrended and bandpass filtered (0.01–0.08 Hz) were performed to remove the effects of very low-frequency drift and high-frequency noise, such as respiratory and heart rhythms. The global mean signal, the white matter signal, the cerebrospinal fluid signal, and the motion parameters (three translational and three rotational parameters) were regressed out. The BOLD signals from each voxel were transformed to a frequency domain by using the Fast Fourier transform (FFT). Then, the power spectrum was square root transformed, and was averaged in the frequency between 0.01 Hz to 0.08 Hz. These resulting amplitudes of the power were taken as the ALFF values. Finally, the ALFF values of all participants were converted into z-scores by subtracting the mean and dividing by the global standard deviation for standardization.

### Statistical analysis

Differences of demographic and clinical variables were per-formed by Pearson χ2 test, one-way analysis of variance (ANOVA), or the Student t-test, as appropriate. A comparison of ALFF maps among h-ET, a-ET, and normal controls was performed by using one-way ANOVA with age, gender and the mean motion parameter as covariates, followed by Tukey-Kramer HSD multiple comparison correction over group pairs. AlphaSim correction was used to correct multiple comparisons over voxels further. The significance threshold was set at p < 0.001 at voxel level and p < 0.05 at cluster level. To assess the relationship between the ALFF value of regions where the three groups showed significant differences and clinical indices of patients with ET, a partial correlation analysis was performed in the patients group to control for the effects of age and gender.
